# Epidemiological study of colovesical fistula as a complication of colonic diverticulitis in Japan: an analysis of claims data

**DOI:** 10.1007/s00595-026-03231-1

**Published:** 2026-01-27

**Authors:** Shunsuke Omotaka, Hiroki Den, Takenori Yamauchi, Ryota Tokunaga, Suguru Ogihara, Masayuki Isozaki, Takahiro Hobo, Noboru Yokoyama, Haruhiro Inoue, Akatsuki Kokaze

**Affiliations:** 1 Department of Hygiene, Public Health, and Preventative Medicine Showa Medical University Graduate School of Medicine, Shinagawa-ku, Tokyo Japan; 2 Digestive Diseases Center, Showa Medical University Koto Toyosu Hospital, Koto-ku, Tokyo Japan

**Keywords:** Colovesical fistula, Colonic diverticulitis, Epidemiology, Claims database

## Abstract

**Purpose:**

Colovesical fistula, a serious complication of colonic diverticulitis, often requires surgical intervention. As the epidemiological data for colovesical fistula associated with colonic diverticulitis in Japan are limited, this study investigated its incidence and characteristics using a claims database.

**Methods:**

This retrospective study analyzed the JMDC Claims Database (January, 2005 to August, 2023). Patients with colonic diverticulitis were identified based on diagnosis codes and concurrent antibiotic treatment. Patients diagnosed with colovesical fistulas who underwent surgery were included in the analysis. Patients with fistulas associated with cancer or inflammatory bowel disease such as ulcerative colitis and Crohn’s disease were excluded.

**Results:**

In total, 42,825 patients with colonic diverticulitis were identified and colovesical fistula developed in 185 (0.43%) of these patients (0.63% in men and 0.07% in women). The median duration from diverticulitis to fistula diagnosis was 0 days, with 73.2% of patients diagnosed simultaneously with, or prior to, diverticulitis. Surgery was performed a median of 49 days after diagnosis.

**Conclusion:**

This first Japanese epidemiological study on colovesical fistulas highlights its low incidence, sex differences, and frequent diagnosis before or with colonic diverticulitis. These findings emphasize the importance of recognizing the diverse clinical presentations for a timely diagnosis and better management.

**Supplementary Information:**

The online version contains supplementary material available at 10.1007/s00595-026-03231-1.

## Introduction

Diverticular disease is common in Western countries [1], and its prevalence is also increasing in Japan [2–4], likely attributable to the westernization of dietary habits. In Japan, the prevalence of colonic diverticula is 23.9% [5], with an average patient age of 52 years. Although patients with colonic diverticula are often asymptomatic [1], this progresses to colonic diverticulitis in 10% to 25% of patients [1, 6], representing the most common complication. In severe cases, abscesses formed by colonic diverticulitis may rupture into adjacent organs, leading to fistula formation [2]. Colovesical fistula, a fistula located between the colon and bladder, is a rare but serious complication of colonic diverticulitis, reported in 2–23% of cases [7–11]. Because of the higher intraluminal pressure in the colon than in the bladder, spontaneous closure is rare, and surgical intervention is often required [2]. Despite its clinical significance, epidemiological studies on colovesical fistulas in Japan remain limited. Although the prevalence of diverticulitis is increasing in Japan, limited research has been conducted on its rare complications, particularly colovesical fistulas.

Medical claims databases are being utilized increasingly for epidemiological research on rare diseases, offering valuable insights into disease incidence and characteristics across large populations [12–17]. We conducted this study to investigate the incidence of colovesical fistula in Japan using a medical claims database, providing important insights into the epidemiology of this rare complication in the Japanese population.

## Methods

1. Data Source

This study utilizes the JMDC Claims Database (JMDC, Inc., Japan), which contains claims data from multiple health insurance societies from January, 2005 to August, 2023. The database links individual patients to their claims through unique patient IDs and claim IDs, ensuring accurate tracking of all medical visits as long as the insurance provider remains unchanged. These IDs are anonymized by the JMDC to prevent the identification of individuals and are not linked to other data sources. Given that the data are primarily from health insurance societies targeting employees of corporations, there is limited representation of individuals aged ≥ 70 years. As of August, 2023, 17,656,319 individuals (9,045,717 men and 8,610,602 women) were registered in the database. Data for each patient were structured using monthly claims records. The database contains basic information, such as date of birth, sex, health insurance enrollment and termination dates, as well as disease names and their corresponding codes, diagnosis dates, and medical procedures performed during the same month.

2. Identification of Patients with Colovesical Fistula Caused by Colonic Diverticulitis

- Colonic Diverticulitis
  Patients with colonic diverticulitis were defined as those who had any of the diagnostic codes listed in Online Resource 1 and received either oral or intravenous antibiotic treatment within the claim records of the same month, for patients registered between January, 2005 and August, 2023. This criterion was chosen to ensure that only patients with a confirmed diagnosis of colonic diverticulitis were included in the analysis, as the use of antibiotics helps differentiate true diverticulitis from suspected cases. This approach is useful for excluding cases where the diagnosis may have been uncertain or not clinically confirmed, such as suspected diverticulitis, which may not require treatment. Only patients aged ≥ 18 years were included in the analysis.
- Patients with Colovesical Fistula Caused by Colonic Diverticulitis
  Data were extracted only for those patients with colonic diverticulitis and a diagnosis code from Online Resource 2 throughout the study period and defined as having a fistula. Patients diagnosed with colovesical fistula, rectovesical fistula, or vesicoenteric fistula were grouped under the term “colovesical fistula” for this study. This grouping was made because these types of fistulas generally share similar clinical management and outcomes, with most requiring surgical intervention because of the higher intraluminal pressure in the colon than in the bladder. Although some reports have suggested that colovesical fistulas may improve with conservative treatment, surgical treatment remains the primary approach for most cases. Therefore, we defined patients with colovesical fistulas caused by colonic diverticulitis as those who had one of these diagnoses and underwent the procedures listed in Online Resource 3, either within the same month or subsequently.
  Patients diagnosed with colorectal cancer, bladder cancer, or inflammatory bowel disease (IBD), either preoperatively or postoperatively (in relation to the surgeries listed in Online Resource 2, assuming a colovesical fistula) and those who had these diagnoses thereafter, were excluded from the study analysis. These cases, which also included a diagnosis of colonic diverticulitis, were considered to have fistula formation caused by cancer or IBD, rather than colonic diverticulitis.

3. Extraction and Analysis Items

The following data on patients with colonic diverticulitis were extracted: date of birth, gender, health insurance enrollment and termination dates, start date of diverticulitis treatment, start date of fistula treatment, date of surgery for colovesical fistula, and the surgical procedure performed. The following analysis items were then calculated: the proportion of patients with colonic diverticulitis who suffered colovesical fistulas stratified by sex and age, age at the time of the first diagnosis of diverticulitis, age at the time of fistula treatment initiation, and age at the time of surgery for colovesical fistulas. Moreover, the duration from the start of diverticulitis treatment (specifically, treatment immediately preceding the diagnosis of colovesical fistula) to the initiation of fistula treatment was calculated. If there was no diverticulitis diagnosis prior to the colovesical fistula diagnosis, the duration from the start of diverticulitis treatment to the fistula diagnosis was used. The duration from the start of fistula treatment to surgery was also calculated.

4. Statistical analysis

Logistic regression analysis was performed to assess temporal trends in the surgical approach, with surgical approach (laparoscopic = 1, open = 0) as the dependent variable and the year of surgery as the independent variable. Odds ratios (ORs) with 95% confidence intervals (CIs) were calculated, and p-values < 0.05 were considered significant. Analyses were conducted for both the entire study period and for patients diagnosed since 2016. Because the number of patients before 2016 was small and not sufficient for stable estimation, the restricted analysis from 2016 onwards was included to ensure the robustness of the findings. All statistical analyses were performed using R software (version 4.1.2; R Foundation for Statistical Computing, Vienna, Austria).

## Results

**Fig. 1:**
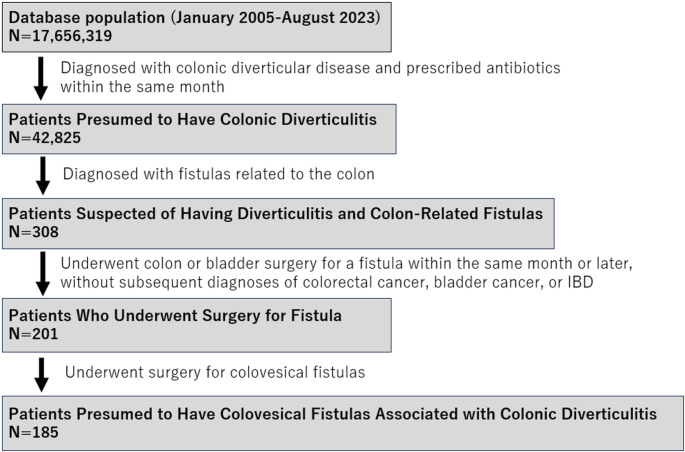
Patient selection flowchart from the initial database population to the final cohort. IBD: inflammatory bowel disease

**Fig. 2:**
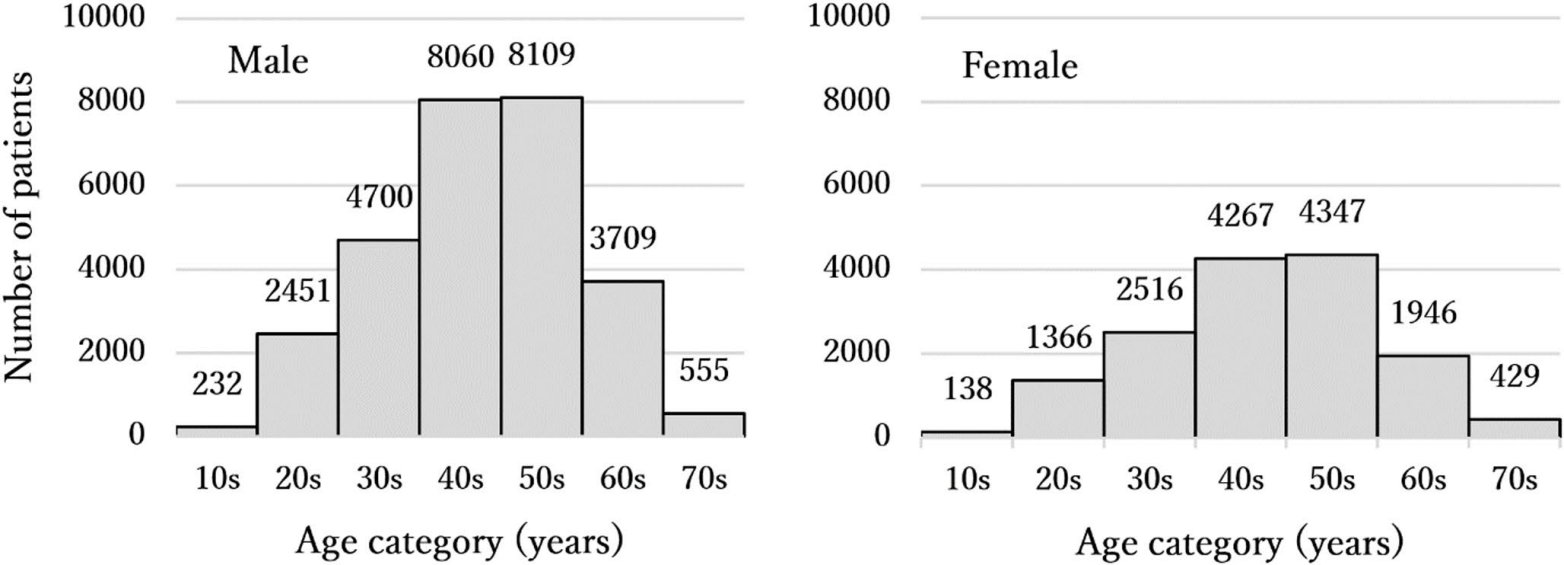
Age distribution of patients with colonic diverticulitis by sex

Figure 1 shows the patient selection flowchart. A total of 42,825 patients with colonic diverticulitis were identified (Fig. 2), with a male-to-female ratio of approximately 2:1. Colovesical fistula was diagnosed in 185 patients (175 men and 10 women). The locations of diverticulitis were as follows: sigmoid colon (*n* = 167), descending colon (*n* = 2), rectum (*n* = 1), and unspecified (*n* = 15) (labeled as “colonic diverticulitis” or " abscessed colonic diverticulitis” without a specific location). According to gender, 158 male and 9 female patients had diverticulitis in the sigmoid colon, 1 male and 1 female patient had diverticulitis in the descending colon, and 1 male and 0 female patients had diverticulitis in the rectum.

Table 1 shows the incidence of colovesical fistulas according to age group. It was more common in men than in women, with the highest incidence observed in patients in their 50 s and 60s. The average age at the time of first diagnosis of colonic diverticulitis in patients who suffered a colovesical fistula was 52 years in men and 56 years in women. The mean age at the time of fistula diagnosis was 57 years for both men and women.

**Table 1:** Number of cases of colovesical fistula among cases of diverticulitis, and complication rates by age group and sex

	Male			Female		
	Number of cases of colovesical fistula	Number of cases of diverticulitis	Complication rate (%)	Number of cases of colovesical fistula	Number of cases of diverticulitis	Complication rate (%)
18–19	0	232	0	0	138	0
20s	1	2451	0.04	0	1366	0
30s	9	4700	0.19	0	2516	0
40s	52	8060	0.65	3	4267	0.07
50s	75	8109	0.92	2	4347	0.05
60s	35	3709	0.94	4	1946	0.21
70s	3	555	0.54	1	429	0.23
Total	175	27,816	0.63	10	15,009	0.07

**Fig. 3:**
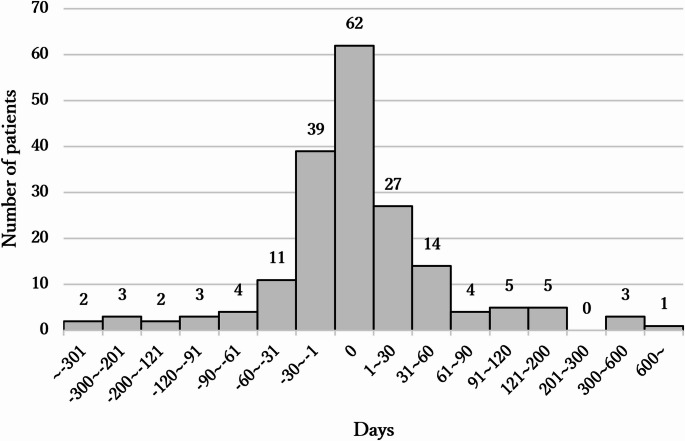
Time interval from the diagnosis of diverticulitis to the diagnosis of fistula

**Fig. 4:**
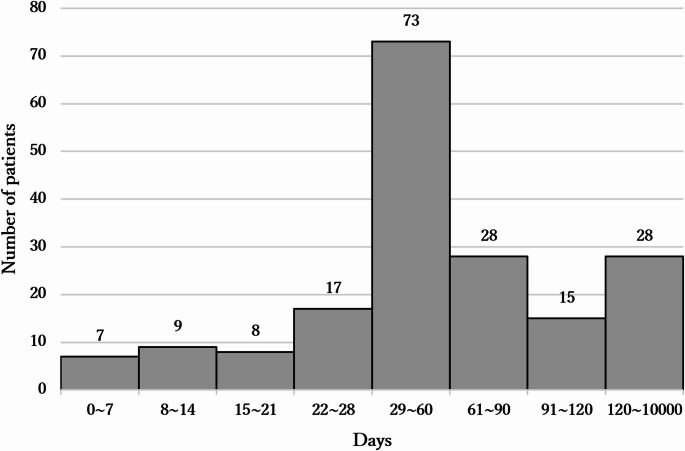
Time interval from the diagnosis of fistula to the date of surgery

Figure 3 shows the period from the diagnosis of diverticulitis to the diagnosis of a fistula. The median was 0 days, and the mean was 25 days. A diagnosis of colovesical fistula was made prior to the diagnosis of colorectal diverticulitis in 64 patients (34.6%), on the same day in 62 (33.5%), and after the diagnosis of colorectal diverticulitis in 59 (31.9%). When limiting the patients affected to those with a period of ± 60 days between the diagnosis of diverticulosis and the diagnosis of fistula, 50 (32.7%) were diagnosed with colovesical fistula before being diagnosed with colorectal diverticulitis, whereas 41 (26.8%) were diagnosed with colovesical fistula after the diagnosis of diverticulitis. Figure 4 illustrates the duration from the diagnosis of colovesical fistula to surgery. The median duration was 49 days (range, 0–3281 days).

**Fig. 5:**
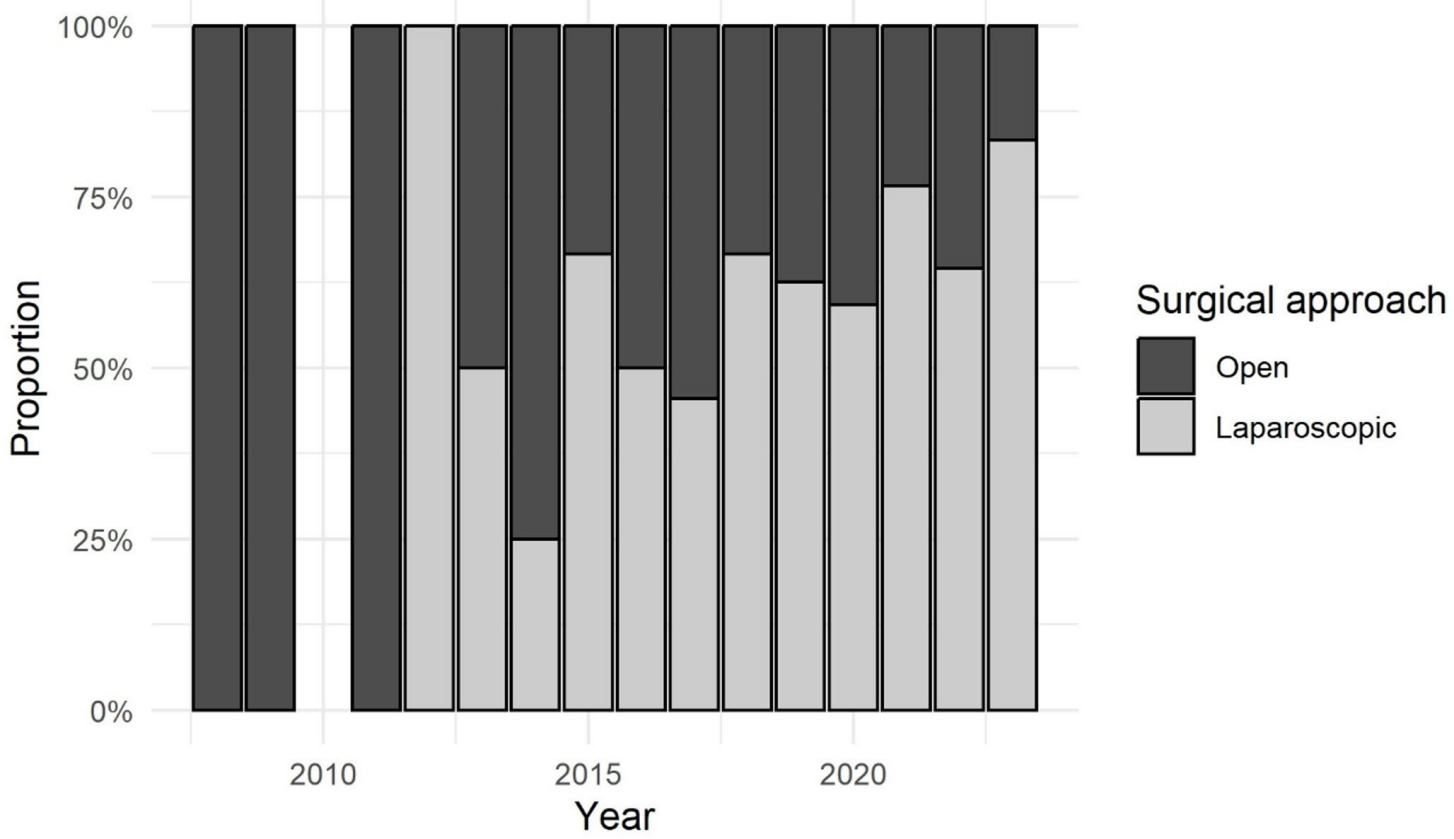
Annual trends in the surgical approach. Stacked bars indicate the proportions of open (black) and laparoscopic (gray) surgeries

Table 2 shows the surgical procedures performed in the 185 patients. Open surgeries were performed in 70 (37.8%) and laparoscopic surgeries were performed in 115 (62.2%). Forty-five patients (24.3%) underwent diverting stoma creation. There were no in-hospital deaths. Figure 5 summarizes the yearly proportions of open and laparoscopic procedures. Logistic regression analysis demonstrated that the proportion of laparoscopic procedures increased significantly over the study period (OR per year = 1.21, 95% CI: 1.09–1.35, *p* < 0.001). When the analysis was restricted to cases since 2016, a consistent increasing trend was also observed (OR per year = 1.19, 95% CI: 1.02–1.40, *p* = 0.029). Other types of fistulas that were surgically treated after diagnosis included enteric fistula (*n* = 1), colonic fistula (*n* = 2), sigmoid colon fistulas (*n* = 4), sigmoid colon-uterine fistulas (*n* = 2), colovaginal fistulas (*n* = 2), rectovaginal fistulas (*n* = 2), rectal fistulas (*n* = 2), and rectocutaneous fistula (*n* = 1).

**Table 2:** Surgical procedures for colovesical fistula

Open surgical procedures (*N*=70)	*N*	Laparoscopic surgical procedures (*N*=115)	*N*
Partial colectomy	45	Laparoscopic colectomy	89
Closure of cystostomy	16	Stoma creation with laparoscopic surgery	29
Stoma creation	16	Laparoscopic rectal resection	10
Hemicolectomy	6	Laparoscopic partial cystectomy	2
Bladder wall resection	5	Laparoscopic total colectomy	1
Low anterior resection	4		

*Totals exceed patient numbers because some patients underwent multiple procedures

## Discussion

This study represents the first epidemiological investigation in Japan of the incidence of colovesical fistulas associated with colonic diverticulitis, using a claims database. The incidence of colovesical fistula was 0.63% in men and 0.07% in women. The highest incidence was of men in their 60 s, with a rate of approximately 1%. The male-to-female ratio for patients with colonic diverticulitis was 2:1, which is consistent with the findings of previous studies [3, 6].

The lower incidence of colovesical fistula in Japan may reflect differences in the healthcare systems and treatment practices. Japan’s universal healthcare system provides affordable access to medical care, enabling the timely and effective management of colonic diverticulitis. Because abscess formation is a critical step in the progression to fistula formation [2], the proactive treatment available in Japan may reduce the likelihood of cases advancing to this stage. The observed sex difference in the incidence of colovesical fistulas among patients with colonic diverticulitis can be attributed to anatomical differences as the female uterus is located between the bladder and the colon [18, 19]. Similar findings regarding this sex difference were reported by Nishimori et al. [20]. Furthermore, the incidence of colovesical fistula was higher in men in the United States [21], and it was also observed more commonly in women who had undergone hysterectomy or in older women with uterine atrophy [18]. None of the women in the present study had undergone hysterectomy. A possible reason for the lower incidence of colovesical fistulas among women in this study than in other reports is the limited representation of individuals aged ≥ 70 years in the study population.

The most common age at diagnosis of colonic diverticulitis was observed in individuals in their 50s. According to Inoue et al., the detection rate of colonic diverticulosis on lower gastrointestinal endoscopy in Japan increased 4.5-fold from the ages of 20 s to the 30 s and continued to rise gradually, reaching 21% in individuals in their 60 s [3]. Given that colonic diverticulitis occurs in 10%–25% of patients with colonic diverticulosis [3], the increasing prevalence of colonic diverticulosis is associated with a higher incidence of colonic diverticulitis. The age of onset of colonic diverticulitis is reported to be in the fourth to sixth decades. The limited number of cases in individuals aged ≥60 years in this study is likely due to the smaller representation of this age group in the study population [5].

Approximately 70% of the colovesical fistulas were diagnosed on the same day or prior to the diagnosis of diverticulitis. Although this finding may seem contradictory in terms of pathophysiology, as diverticulitis typically precedes the development of a colovesical fistula, it is consistent with reports of patients presenting with urinary symptoms such as dysuria, rather than gastrointestinal symptoms such as abdominal pain [7, 11, 22], when seeking medical attention for a colovesical fistula caused by diverticulitis. Fujita et al. reported that 44% of their patients with colovesical fistula caused by diverticulitis did not present with gastrointestinal symptoms, including abdominal pain [2]. It is likely that these patient did not seek medical attention for diverticulitis initially but rather visited the hospital for urinary symptoms. Thus, a detailed medical history should be obtained for patients presenting with urinary symptoms. If a colovesical fistula is suspected, further investigation is necessary. In clinical practice, diagnosis is often based on a combination of imaging findings, such as bladder or bowel wall thickening and the presence of gas within the bladder on CT scans, along with findings from cystoscopy [23, 24].

Manabe et al. reported that the average hospitalization duration for complicated diverticulitis was 24.1 ± 19.5 days [25]. If a colovesical fistula is diagnosed within 60 days of diverticulitis diagnosis, it is considered that the colovesical fistula developed during the diverticulitis treatment period. Conversely, if a colovesical fistula is diagnosed > 60 days before or after the diagnosis of diverticulitis, it is possible that these cases may have involved missed coding of the diagnosis or that the diagnosis was not officially discontinued and then continued when diverticulitis recurred. In this study, there was no information available for periods not registered in the JMDC claims data, so it is impossible to determine whether diverticulitis was the first occurrence or, in cases of recurrence, how many times it had recurred. There may be no significant difference in the incidence of colovesical fistulas between the first occurrence and the recurrence of diverticulitis [2]. In clinical practice, it is difficult to accurately determine whether the number of recurrences and whether the diverticulitis is a first occurrence or a recurrence.

Tomizawa et al. reported that the median age at the time of surgery for colovesical fistulas was 60 years [20], which may be attributable to the small proportion of older individuals in the population. Regarding the duration from the diagnosis of a colovesical fistula to surgery in this study, 49 patients (26.5%) underwent surgery within 1 month of diagnosis, whereas 65 (35.1%) underwent surgery 1–2 months after diagnosis. The remaining patients (38.4%) underwent surgery after this period. Urinary tract infections temporarily improve with conservative treatment. Therefore, emergency surgery is not typically performed. In recent years, the proportion of laparoscopic abdominal procedures has been increasing [26], and for some surgeries, laparoscopic approaches now outnumber open approaches [27]. Accordingly, the rate of laparoscopic procedures for colovesical fistulas caused by colonic diverticulitis is also presumed to be rising.

Previous reports indicate that the rate of stoma creation during surgeries for colovesical fistulas ranges from 0% to 12.3% [19, 20], which is lower than the rate observed in our study. One possible explanation is that institutions reporting large case volumes may have greater experience and confidence in performing resections without stoma creation, whereas smaller institutions may adopt a more conservative surgical approach. This variation could contribute to the higher overall rate of stoma creation observed in our dataset. Moreover, previous reports have recommended stoma creation for patients with peritonitis or sepsis, extensive adhesions, advanced age, or when significant tension is expected at the anastomotic site [20, 28, 29]. However, this study could not assess the clinical factors associated with stoma creation because such detailed information is not available in claims data. Future studies using detailed clinical data will be required to clarify this.

A limitation of this study is that we defined patients with colovesical fistula as those who had specific disease codes in the claims database and underwent surgery. However, some patients may have opted against surgical intervention because of their own treatment choices or overall health status. Takenaga et al. reported that colovesical fistulas may improve with conservative management [24], suggesting that the number of colovesical fistula cases may have been underestimated in this study. Nevertheless, given that spontaneous closure and healing of colovesical fistulas is rare, the impact of this underestimation of the overall results is probably minimal. Conversely, some patients who were classified as having a colovesical fistula may have undergone surgery despite the absence of a fistula, potentially leading to an overestimation of cases. Furthermore, some surgical procedures may have been performed for colonic perforation or strictures secondary to diverticulitis rather than for the colovesical fistula. However, we identified patients who underwent surgery after the diagnosis of a colovesical fistula. Although surgical intervention for colonic perforation is typically performed on an emergency basis, only a limited number of patients underwent surgery immediately after a colovesical fistula was diagnosed. Therefore, we do not believe that this factor had a significant impact on the validity of our findings. Another limitation of this study was the follow-up duration in the claims database, with a median follow-up period of 3 years 9 months (range: 1 month–18 years 8 months). Following changes in insurance coverage associated with job transitions or marital status, some patients had relatively short observation periods before and after the onset of diverticulitis or colovesical fistulas. Consequently, it was not always possible to identify the precise age at the initial diagnosis of diverticulitis or the duration from the onset of diverticulitis to the development of a colovesical fistula. Moreover, the study population underrepresented individuals aged ≥70 years, who are at higher risk of complications such as colovesical fistula. This underrepresentation limits the generalizability of our findings to older adults, warranting cautious interpretation while applying these results to this population.

In conclusion, this study provides important insights into the epidemiology of colovesical fistulas associated with colonic diverticulitis in Japan. The incidence of colovesical fistulas was significantly higher in men than in women (0.63% vs. 0.07%, respectively), particularly in those in their 50 s and 60s. These findings suggest that the proactive treatment of diverticulitis in Japan, supported by the universal healthcare system, may contribute to a lower incidence of this severe complication. Although colovesical fistulas originate from colonic diverticular disease, patients often present with urological rather than gastrointestinal symptoms. This finding underscores the clinical importance of considering colovesical fistulas as an underlying cause in patients with recurrent or refractory urinary tract infections to facilitate timely diagnosis and management. However, due to the limited representation of individuals aged ≥70 years in the claims database, the generalizability of these findings to older populations remains uncertain. Further studies are required to investigate the incidence and clinical management of colovesical fistulas in older patients.

## Supplementary Information

Below is the link to the electronic supplementary material.

Supplementary Material 1

Supplementary Material 2

Supplementary Material 3

## References

[1] Place RJ, Simmang CL. Diverticular disease. Best Pract Res Clin Gastroenterol. 2002;16:135–48. 11977933 10.1053/bega.2001.0270

[2] Fujita Y, Koda K. A clinical study on cases of colovesical fistula caused by diverticulitis(in Japanese with english abstract). Nihon Daityoukoumonnbyou Kaishi (J Jpn Soc Coloproctol). 2014;67:442–7.

[3] Inoue M. Epidemiology and clinical features of colonic diverticular disease(in Japanese). Nihon Daichoukoumonbyou Kaishi(J Jpn Soc Coloproctol). 1992;45:904–13.

[4] Sadahiro Y, Suzuki T, Maeda Y, Tanaka A, Iwase H. Pathophysiology of diverticular disease of the colon(in Japanese with english abstract). Nihon Daityoukoumonnbyou Kaishi(J Jpn Soc Coloproctol). 2008;61:1016–20.

[5] Fujimoto K, Kaise M, Iwai N, Urita S, Tomizawa K, Nagata N, et al. Guidelines for colonic diverticular bleeding and colonic diverticulitis(in Japanese). Tokyo: The Japanese Gastroenterological Association; 2017. pp. 5–6.

[6] Hanna MH, Kaiser AM. Update on the management of sigmoid diverticulitis. World J Gastroenterol. 2021;27:760–81. 33727769 10.3748/wjg.v27.i9.760 PMC7941864

[7] Yang HY, Sun WY, Lee TG, Lee SJ. A case of colovesical fistula induced by sigmoid diverticulitis. J Korean Soc Coloproctol. 2011;27:94–8. 21602969 10.3393/jksc.2011.27.2.94 PMC3092082

[8] Melchior S, Cudovic D, Jones J, Thomas C, Gillitzer R, Thüroff J. Diagnosis and surgical management of colovesical fistulas due to sigmoid diverticulitis. J Urol. 2009;182:978–82. 19616793 10.1016/j.juro.2009.05.022

[9] Najjar SF, Jamal MK, Savas JF, Miller TA. The spectrum of colovesical fistula and diagnostic paradigm. Am J Surg. 2004;188:617–21. 15546583 10.1016/j.amjsurg.2004.08.016

[10] Solkar MH, Forshaw MJ, Sankararajah D, Stewart M, Parker MC. Colovesical fistula - is a surgical approach always justified? Colorectal Dis. 2005;7:467–71. 16108883 10.1111/j.1463-1318.2005.00863.x

[11] Xu R, Vaughan A, Fagan M, Schumacher DP, Wekullo V, Gehrke B. Colovesical fistula in men with chronic urinary tract infection: a diagnostic challenge. Cleve Clin J Med. 2023;90:165–71. 36858611 10.3949/ccjm.90a.21060

[12] Den H, Ito J, Kokaze A. Epidemiology of developmental dysplasia of the hip: analysis of Japanese National database. J Epidemiol. 2023;33:186–92. 34380918 10.2188/jea.JE20210074 PMC9939923

[13] Hiramatsu A, Den H, Morita M, Ogawa Y, Fukagai T, Kokaze A. A nationwide epidemiological study of testicular torsion: analysis of the Japanese National database. PLoS One. 2024;19:e0297888. 38457468 10.1371/journal.pone.0297888 PMC10923415

[14] Nakashima M, Takeuchi M, Kawakami K. Clinical outcomes of acute appendicitis during pregnancy: conservative management and appendectomy. World J Surg. 2021;45:1717–24. 33635341 10.1007/s00268-021-06010-w

[15] Fujimoto S, Tsuruoka N, Esaki M, Takamori A, Sakata Y, Shimoda R, et al. Decline incidence in upper gastrointestinal bleeding in several recent years: data of the Japan claims database of 13 million accumulated patients. J Clin Biochem Nutr. 2021;68:95–100. 33536718 10.3164/jcbn.20-153 PMC7844659

[16] Yoshida N, Maeda-Minami A, Ishikawa H, Mutoh M, Kanno Y, Tomita Y, et al. Analysis of the development of gastric cancer after resecting colorectal lesions using large-scale health insurance claims data. J Gastroenterol. 2023;58:1105–13. 37646980 10.1007/s00535-023-02035-1

[17] Matsuoka K, Togo K, Yoshii N, Hoshi M, Arai S. Incidence rates for hospitalized infections, herpes zoster, and malignancies in patients with ulcerative colitis in Japan: an administrative health claims database analysis. Intest Res. 2023;21:88–99. 35263962 10.5217/ir.2021.00154 PMC9911274

[18] Nishimori H, Hirata K, Fukui R, Sasaki M, Yasoshima T, Nakajima F, et al. Vesico-ileosigmoidal fistula caused by diverticulitis: report of a case and literature review in Japan. J Korean Med Sci. 2003;18:433–6. 12808335 10.3346/jkms.2003.18.3.433 PMC3055041

[19] Murakami T, Matsui Y, Ohyama A, Mizuno R, Ohkoshi Y, Honma S. Five cases of colovesical fistula due to diverticulitis treated by laparoscopic surgery(in Japanese with english abstract). Nihon Rinsho Geka Gakkai Zasshi (J Jpn Surg Assoc). 2021;82:1051–6.

[20] Tomizawa K, Toda S, Tate T, Hanaoka Y, Moriyama J, Matoba S, et al. Laparoscopic surgery for colovesical fistula associated with sigmoid colon diverticulitis: a review of 39 cases. J Anus Rectum Colon. 2019;3:36–42. 31559365 10.23922/jarc.2018-008 PMC6752128

[21] Keller-Biehl L, Yu KR, Smith-Harrison L, Timmerman W, Rivers J, Miller T. Colovesical fistula: a 28-year experience at a major United States Department of Veterans Affairs Medical Center. Surg Pract Sci. 2022;11:100100. 39845169 10.1016/j.sipas.2022.100100 PMC11749922

[22] Lavery IC. Colonic fistulas. Surg Clin North Am. 1996;76(5):1183–90. 10.1016/s0039-6109(05)70506-5 8841372

[23] Rahman M, Tokunaga S, Ikeda D, Yokoyama O, Ohkawa M, Fujita H, et al. Colovesical fistula due to sigmoid colon diverticulitis: a case report. Hinyoukika Kiyou (Acta Urol Jpn). 1995;41:231–4. 7741079

[24] Takenaga M, Hosokawa Y, Iida K, Itami Y, Shinohara M, Hayashi M, et al. Non-surgical treatment of vesico-sigmoid fistula secondary to diverticulitis:aacase report(in Japanese with english abstract). Hinyoukigeka (Jpn J Urol Surg). 2014;27:233–6.

[25] Manabe N, Haruma K, Nakajima A, Yamada M, Maruyama Y, Gushimiyagi M, et al. Characteristics of colonic diverticulitis and factors associated with complications: a Japanese multicenter, retrospective, cross-sectional study. Dis Colon Rectum. 2015;58:1174–81. 26544815 10.1097/DCR.0000000000000488

[26] Takahashi T, Nishiura H. Increasing rates of laparoscopic gastrointestinal surgery and decreasing rates of surgical site infections: an observational study in Japan from 2012–2017. BMC Surg. 2021;21:370. 10.1186/s12893-021-01373-2. 34670525 10.1186/s12893-021-01373-2 PMC8527652

[27] Yamamoto T, Takahashi A, Yoshizumi T, Ishihara S, Inomata M, Imoto S et al. 2023 National Clinical Database Annual Report by the Japan Surgical Society. Surg Today. 2025;55:295–334. 10.1007/s00595-024-02980-1 PMC11842510 39820660

[28] Marney LA, Ho YH. Laparoscopic management of diverticular colovesical fistula: experience in 15 cases and review of the literature. Int Surg. 2013;98:101–9. 10.9738/INTSURG-D-13-00024.1 PMC3723180 23701143

[29] Smeenk RM, Plaisier PW, van der Hoeven JAB, Hesp WLEM. Outcome of surgery for colovesical and colovaginal fistulas of diverticular origin in 40 patients. J Gastrointest Surg. 2012;16:1559–65. 22653331 10.1007/s11605-012-1919-1

