# Supplementary material for: Epidemiological study of colovesical fistula as a complication of colonic diverticulitis in Japan: an analysis of claims data
Source: Surg Today. 2026 Jan 27;56(7):1258–66. doi: 10.1007/s00595-026-03231-1 (PMC13303533; doi:10.1007/s00595-026-03231-1)
Supplement: Supplementary file 1 — Supplementary Material 1 [file 595_2026_3231_MOESM1_ESM.docx]

Online Resource 1: List of Diverticular Diseases and Corresponding Japanese Standard Disease Codes and ICD-10 Codes

| **Name of Diverticular Diseases** | **Japanese Standard**  **Disease Codes** | **ICD-10**  **Codes** |
| --- | --- | --- |
| sigmoid colon diverticulum | 8830140 | K573 |
| sigmoid colon diverticulitis | 8845741 | K573 |
| multiple sigmoid colon diverticula | 8845744 | K573 |
| sigmoid colon diverticulum perforation | 8845743 | K572 |
| sigmoid colon diverticulitis with abscess | 8838773 | K572 |
| colon diverticulosis | 8833154 | K573 |
| colon diverticulitis | 5621008 | K573 |
| colon diverticulum perforation | 8845781 | K572 |
| colon diverticulitis with abscess | 8838776 | K572 |
| multiple colon diverticula | 8837055 | K573 |
| rectum diverticulitis | 8845819 | K573 |
| rectum diverticulum | 5621025 | K573 |
| rectum diverticulum perforation | 8847528 | K572 |
| ascending colon | 8838777 | K572 |
| descending colon | 8838775 | K572 |
| transverse colon | 8838774 | K572 |
| transverse colon diverticulum perforation | 8845750 | K572 |
| descending colon diverticulum perforation | 8845764 | K572 |
| ascending colon diverticulum perforation | 8845807 | K572 |
| descending colon diverticulitis | 8845762 | K573 |
| transverse colon diverticulitis | 8845748 | K573 |
| ascending colon diverticulitis | 8845805 | K573 |
| multiple ascending colon diverticula | 8845808 | K573 |
| multiple descending colon diverticula | 8845765 | K573 |
| multiple transverse colon diverticula | 8845751 | K573 |
| transverse colon diverticulum | 8830960 | K573 |
| descending colon diverticulum | 8831198 | K573 |
| ascending colon diverticulum | 8835409 | K573 |
