# Supplementary material for: Epidemiological study of colovesical fistula as a complication of colonic diverticulitis in Japan: an analysis of claims data
Source: Surg Today. 2026 Jan 27;56(7):1258–66. doi: 10.1007/s00595-026-03231-1 (PMC13303533; doi:10.1007/s00595-026-03231-1)
Supplement: Supplementary file 2 — Supplementary Material 2 [file 595_2026_3231_MOESM2_ESM.docx]

Online Resource 2: List of Fistula Diseases and Corresponding Japanese Standard Disease Codes and ICD-10 Codes

| **Name of Fistula Diseases** | **Japanese Standard**  **Disease Codes** | **ICD-10**  **Codes** |
| --- | --- | --- |
| colovesical fistula | 5961010 | N321 |
| colon fistula | 8833160 | K632 |
| vesicoenteric fistula | 5961005 | N321 |
| sigmoid colonic endometriosis | 8845206 | N825 |
| sigmoid colon fistula | 8830144 | K632 |
| rectovesical fistula | 5961003 | N321 |
| rectum fistula | 5651014 | K604 |
| rectovaginal fistula | 8837788 | N823 |
| colovaginal fistula | 8837376 | N823 |
| gastrocolic fistula | 8830429 | K316 |
| rectocutaneous fitula | 8837792 | ML39 |
| cholecystocolonic fistula | 8837171 | H83H |
| enteric fistula | 8837772 | K632 |
| colovaginal fistula | 8837752 | N824 |
