# Supplementary material for: Epidemiological study of colovesical fistula as a complication of colonic diverticulitis in Japan: an analysis of claims data
Source: Surg Today. 2026 Jan 27;56(7):1258–66. doi: 10.1007/s00595-026-03231-1 (PMC13303533; doi:10.1007/s00595-026-03231-1)
Supplement: Supplementary file 3 — Supplementary Material 3 [file 595_2026_3231_MOESM3_ESM.docx]

Online Resource 3: List of Surgical Procedures and Corresponding Japanese Standard Procedure Codes

| **Surgical procedures** | **Japanese Standard  Procedure Codes** |
| --- | --- |
| partial colectomy | 150181710 |
| hemicolectomy | 150181810 |
| stoma creation surgery | 150184510 |
| colostomy | 150184510 |
| proctectomy | 150187110 |
| bladder wall resection | 150199210 |
| cystectomy | 150199610 |
| laparoscopic colectomy | 150277810 |
| rectal resection/amputation (ultra-low anterior resection) | 150297510 |
| laparoscopic rectal resection | 150325210 |
| laparoscopic total colectomy | 150337710 |
| laparoscopic low anterior resection | 150337810 |
| laparoscopic partial cystectomy | 150379210 |
| laparoscopic stoma creation surgery | 150389610 |
| closure of cystostomy(endoscopy) | 150404110 |
| closure of cystostomy | 150404210 |
| stoma creation addition(colectomy) | 150420070 |
| stoma creation addition  (laparoscopic colectomy) | 150420170 |
| stoma creation addition (laparoscopic rectal resection/amputation surgery)  (resection surgery) | 150440570 |
| low anterior resection | 150440970 |
| stoma creation addition (laparoscopic rectal resection/amputation) (ultra-low anterior resection) | 150441070 |
